# Supplementary material for: A full-body transcription factor expression atlas with completely resolved cell identities in C. elegans
Source: Nat Commun. 2024 Jan 9;15:358. doi: 10.1038/s41467-023-42677-6 (PMC10776613; doi:10.1038/s41467-023-42677-6)
Supplement: Supplementary file 1 — Supplementary Information [file 41467_2023_42677_MOESM1_ESM.pdf]

Supplementary Material for

A full-body transcription factor expression atlas with completely resolved cell identities in *C. elegans*

Yongbin Li[1,2,6], Siyu Chen[2,6], Weihong Liu[2,3,6], Di Zhao[2,4,6], Yimeng Gao[1,6], Shipeng Hu[1], Hanyu Liu[1], Yuanyuan Li[5], Lei Qu[5], and Xiao Liu[1]*

[1]College of Life Sciences, Capital Normal University, Beijing 100048, China. [2]School of Life Sciences, Tsinghua University, Beijing 100084, China. [3]Intelligent Perception Lab, Hanwang Technology Co., Ltd, 100193 Beijing, China. [4]Tianjin Key Laboratory of Exercise Physiology and Sports Medicine, Institute of Sport, Exercise & Health, Tianjin University of Sport, Tianjin 300381, China. [5]Ministry of Education Key Laboratory of Intelligent Computation & Signal Processing, Information Materials and Intelligent Sensing Laboratory of Anhui Province, School of Electronics and Information Engineering, Anhui University, Hefei 230039, China. [6]These authors contributed equally: Yongbin Li, Siyu Chen, Weihong Liu, Di Zhao, Yimeng Gao. Correspondence and requests for materials should be addressed to X. L. (E-mail: liux@cnu.edu.cn)

This PDF file includes:

Supplementary Note

Supplementary Figures 1-8

Supplementary Table 1

Supplementary Note

Rationale for the 5-template annotation

At the first thought, there should be a single template corresponding to the stereotypic worm body, which essentially has invariable cell lineage and positions [1]. However, variations in developmental program have been well-documented to result in slight variations in the exact location of cell nuclei [1, 2, 3]. Furthermore, cell identity can determine potential variability of developmental program for individual cells. For example, a ring of four nuclei is invariably found surrounding the lumen in the anterior-most region of the intestine [1]. By contrast, the arrangement of neurons anterior to the intestine shows relatively high variability among newly hatched L1 larvae [2]. In addition, worms adopt different curvatures at the time of fixation for confocal imaging. As a result, their 3D image stacks require computational straightening so that they can be annotated by a canonical coordinate system. The straightening process could potentially deform relative cell locations [4]. In short, several factors, such as cell identity-related differences in developmental program, and artifacts of the image straightening process for curved worm bodies can collectively contribute slight differences in cell positions among stereotype templates.

In practice, the 5-template-based annotation showed higher accuracy and more robust annotations compared to the optimal-template-based results in tests of 100 image stacks (Figure 1F), thus supporting our intuition that multiple templates can better capture inter-prototype variation in the positions of cell nuclei. However, the variation in mean and variance of cell positions among different templates was notably smaller than the distances between neighbor cells (Supplementary Figures 2E and 2F). Despite being subtle, these differences in mean and variance of cell positions between templates led to distinguishable matching scores (Supplementary Figures 2G and 2H), justifying the use of separate templates for assigning cell identities.

Validation of accuracy of RAPCAT using published TF profile in adult nervous systems

Digital templates and RAPCAT annotation totally depends on relative positions of cells. On the contrary, neuron identifications with NeuroPAL landmarks are likely more accurate because they utilized both neuron positions and well-established markers[3]. Expression of 101 homeodomain TF had been profiled with individual neuron resolution in adult nervous system using NeuroPAL [5]. These data can be gold standards to test the accuracy of our RAPCAP annotation.

We obtained 11 of these TF reporters from colleagues in the worm research community. Among the 222 neurons already present in L1 larvae, these reporters were expressed in 1 – 108 neurons of young adults (Supplementary Figure 4E). We generated image stacks of the 11 reporter strains and annotated them using RAPCAT without the manual EPC step (*i.e.,* the stacks were annotated automatically). One stack, capturing *hmbx-1* expression, did not pass the 0.995 WAC threshold and was therefore excluded from the validation of automated RAPCAT annotation accuracy. For the remaining 10 reporters, RAPCAT annotation indicated that a TF was simultaneously expressed in at least 1 and as many as 80 neurons. Among the 222 total neurons, 140 showed detectable GFP expression by at least one reporter at the L1 stage in our 10-TF profile. Among the 140 GFP-expressing neurons in our results, 128 were also positive for expression of all corresponding reporters in young adults (Supplementary Figure 4E), suggesting 91.4% consistency between the automated neuron annotation by RAPCAT and marker-based identity assignment by NeuroPAL.

Transcriptomic changes have been previously reported in the post-mitotic nervous system during larval development [6]. It is thus reasonable to speculate that some discrepancies between RAPCAT annotation based on L1 expression profile and the previously published adult profile may be due to temporal changes in gene expression

rather than error in RAPCAT annotation. We conducted a manual examination of the RAPCAT annotation outcomes for the 12 neurons that exhibited detectable GFP expression exclusively in L1 larvae, without the same expression observed in young adults. Based on our thorough inspection, it was determined that seven out of these 12 neurons had received accurate cell identity assignment through the RAPCAT annotation process. In other words, automatic RAPCAT annotation errors were present in only five neurons. These included a tail neuron PHB, a pharyngeal neuron I6, and three neurons (ASGL, AVDL, and AVHR) located around the nerve ring (Supplementary Figure 4E). Collectively, the automatic RAPCAT annotation method effectively and unambiguously identifies neurons in L1 larvae, achieving an accuracy ranging from 91.4% to 96.4%, encompassing even the densely packed neurons in the brain.

The impact of technical limitations on TF-base cell clustering

We conducted an analysis to measure the alignment between traditional cell classifications and TF-driven cell clustering based on TF profiles with various technical limitations. In the hierarchical clustering dendrogram based on our final TF profiles, fourteen out of 119 multi-cellular phenotypic cell classes were distributed across multiple clades (Figure 3 and Supplementary Data 6). This distribution pointed to disparities between phenotype-based classification and molecular clustering, specifically relying on the profiles that encompassed all 620 TFs examined in the current study and whose RAPCAT annotation was in the mode with the highest accuracy.

The cell annotation accuracy of the discordant cell classes was significantly lower than that of the concordant cell classes (Supplementary Figure 6J), suggesting a potential impact of annotation error on cell clustering. In order to quantify this impact, we generated TF profiles using each RAPCAT mode, with annotation accuracy rates ranging from 88% to 97% based on analysis of the second set of 100 testing stacks

(Figure 1H). Subsequently, we clustered cells using these profiles. The effect of annotation accuracy on the agreement between phenotype-based classification and molecular clustering was marginal when annotation accuracy ranged from 93% to 97% (Supplementary Figure 6K). Further exploration involved clustering cells based profiles containing varying numbers of TFs, revealing that the alignment between phenotype-based classification and molecular clustering was closely linked to the size of the TF dataset. Notably, the impact of the number of TFs on the ability of molecular clustering to mirror established phenotype-based cell type classifications reached a plateau when the profiles contained over 400 TFs (Supplementary Figure 6K). For instance, even upon excluding the 61 fosmid/knock-in reporters from the 620-TF profiles, the number of concordance cell classes between phenotype-based classification and molecular clustering remained unaffected (Supplementary Figure 6K).

As described in the main text, we categorized the reporters of the 620 profiled TFs into three distinct types based on the extent to which their reporter constructs encompassed regulatory elements (Figure 2C). These categorized comprised 61 knock-in/fosmid reporters, 296 high-context promoter reporters, and 263 low-context promoter reporters (Supplementary Data 1). Upon conducting further analysis and comparing the results with previously published homeobox TF profiles in young adults [5], it became evident that both knock-in/fosmid and high-context promoter reporters effectively captured the comprehensive expression profiles of their respective genes. In contrast, the low-context promoter reporters demonstrated relatively weaker correlations with the published adult stage profiles (Figure 2C).

Upon excluding the 263 low-context promoter reporters from our profiles, additional six cell classes exhibited discordance between phenotype-based classification and TF-based clustering (Supplementary Figure 6L). This suggests that low-context promoter reporters could potentially offer insights into the gene regulation program sensed by

specific DNA sequences, even if they did not entirely encompass the expression patterns of their corresponding endogenous genes. Notably, when we excluded reporters that were likely comprehensive in capturing the complete expression profiles of their respective genes (specifically, all 61 fosmid/knock-in reporters and a random selection of 202 high-context promoter reporters), it is intriguing that the count of cell classes exhibiting discordance between phenotype-based classification and TF-based clustering remained comparable to that observed when profiles excluding the 263 low-context promoter reporters were considered (18 *vs*. 20) (Supplementary Figure 6L).Moreover, we found molecular clustering of the large set of 263 low-context reporters could recapitulate well-established phenotype-based cell type classifications to strikingly higher extent than a smaller set of 61 fosmid/knock-in reporters (Supplementary Figure 6K). This finding led us to conclude that the number of profiled genes affected TF-based cell type identification more strongly than full genomic context.

TF expression corresponds with morphological heterogeneity within cell types

Our TF profiling classified pharyngeal muscles into four subtypes (pm1-pm2, pm3-pm5, pm6-pm7, and pm8) (Supplementary Figure 6E). Specifically, pm1-pm2 cells have small soma, thin processes, and form rings anteriorly, while other pharyngeal muscles have large soma and radially oriented contractile filaments [7] (Supplementary Figure 6H). Correspondingly, the pm1-pm2 cells show negligible expression of pharynx-specific the myosin heavy chain gene *myo-2* [8], strongly expressed in the tonic pm3-pm8 muscles (Supplementary Figure 6I). Pharyngeal epithelial cells formed the neighbor-clade of the pm1-pm2 subtype (Supplementary Figure 6E), and share a similar morphology with pm1-pm2 cells [7].

Our findings of heterogeneity also showed concordance between TF profiling and morphology in hypodermis, which were classified into two subtypes: hyp1-3 and hyp4-

11. While hyp4-11 cells exhibit a sheet-like morphology to envelope the worm body, hyp1-3 cells form concentric rings that outline the anterior-most edge of the hypodermis[9]. Consistent with their morphological and functional differences, hyp1-3 cells expressed three hypodermal genes (*elt-3*, *dpy-7*, and *col-93*) at lower levels than that in hyp 4-11 cells (Supplementary Figure 6K). The GATA family TF, ELT-3, specifies hypodermal cell fate [10] while the collagen proteins DPY-7 and COL-93 are components of the cuticle [11, 12]. The neighbor-clade to the hyp4-11 subtype was composed of P neuroblasts (Supplementary Figure 6F). Although P neuroblasts are neural precursor cells, they also have sheet-like morphology and act as the ventral hypodermis at the L1 stage [1].

Concordance between TF profiling and morphology was observed in neuroblasts too. Our TF-based subtyping identified four neuroblast subtypes, including P, Q/V5/T, G1/G2/W, and K (Figure 4C). Although P and W neuroblasts have highly related postembryonic cell lineage to give rise to neurons while they have very different morphology[1], they were classified into different subtypes based on TF profiling, consistent with their morphological heterogeneity (Figure 4C). Instead, the neighbor-clade of P neuroblasts was composed of hypodermis hyp7, both of which are large epithelial cells covering the main body of worms [9]. The G1 and G2 neuroblasts act as transient excretory pore cells before and after embryo hatching [13]. Although the W neuroblast has different morphology in an intact embryo, it can become G2 neuroblast when the G2 cell is ablated [2]. Excretory pore cells and the excretory duct cell are both specialized epithelial cells wrapping around the excretory duct. Consistent with their similar morphology and function, the G1/G2/W and excretory duct cells are neighbor-clades of each other based on TF profiling (Figure 4C). Altogether, TF expression profiles of P and G1/G2/W neuroblasts are more associated with their morphology than with their post-embryonic developmental programs.

On either the left or right side of the worm body at the early L1 stage, there is a longitudinal row of seam cells, composed of neuroblasts (V5 and T), hypodermal precursor cells (H, V1-V4, and V6), and non-proliferative H0 cell[1]. All seam cells are smoothly tapered in shape [9] (Figure S6J). Correspondingly, the neighbor-clade cells of TF-based Q/V5/T subtype were non-neuroblast seam cells (Figure 4C). At last, the K neuroblast and the K' epithelial cell are a pair of symmetric cells so that they have identical morphology[1]. They are neighbor-clades of each other based on TF profiling too (Figure 4C).

In summary, subtype classification based on TF profiling is consistent with intra-type morphological heterogeneity. Moreover, although a TF-based subtypes are different cell types than their respective neighbor clades, they share remarkably similar morphology.

Rationale for identification of neuroblast subtypes sharing the same pro-neural TF battery

Different TF batteries underlie morphological varieties of epithelial subtypes. Neuroblasts can be considered as special epithelial cells expressing proneural TF batteries. Different pro-neural TF batteries underlie various neural developmental programs. As such, differences in complete TF profiles between neuroblast subtypes are composed of differences in their morphological TF batteries and pro-neural TF batteries. For neuroblast subtypes driven by the same pro-neural battery, their differences in their complete TF profiles can be large if they have dramatically different morphological TF batteries. If we manage to exclude differences in morphological TF batteries, molecular similarity between neuroblast subtypes driven by same pro-neural TF battery can be revealed. Excluding the difference in morphological TF batteries was computationally achieved by normalization of their cross-neighbor clade difference in JSD score (Supplementary Figure 5D).

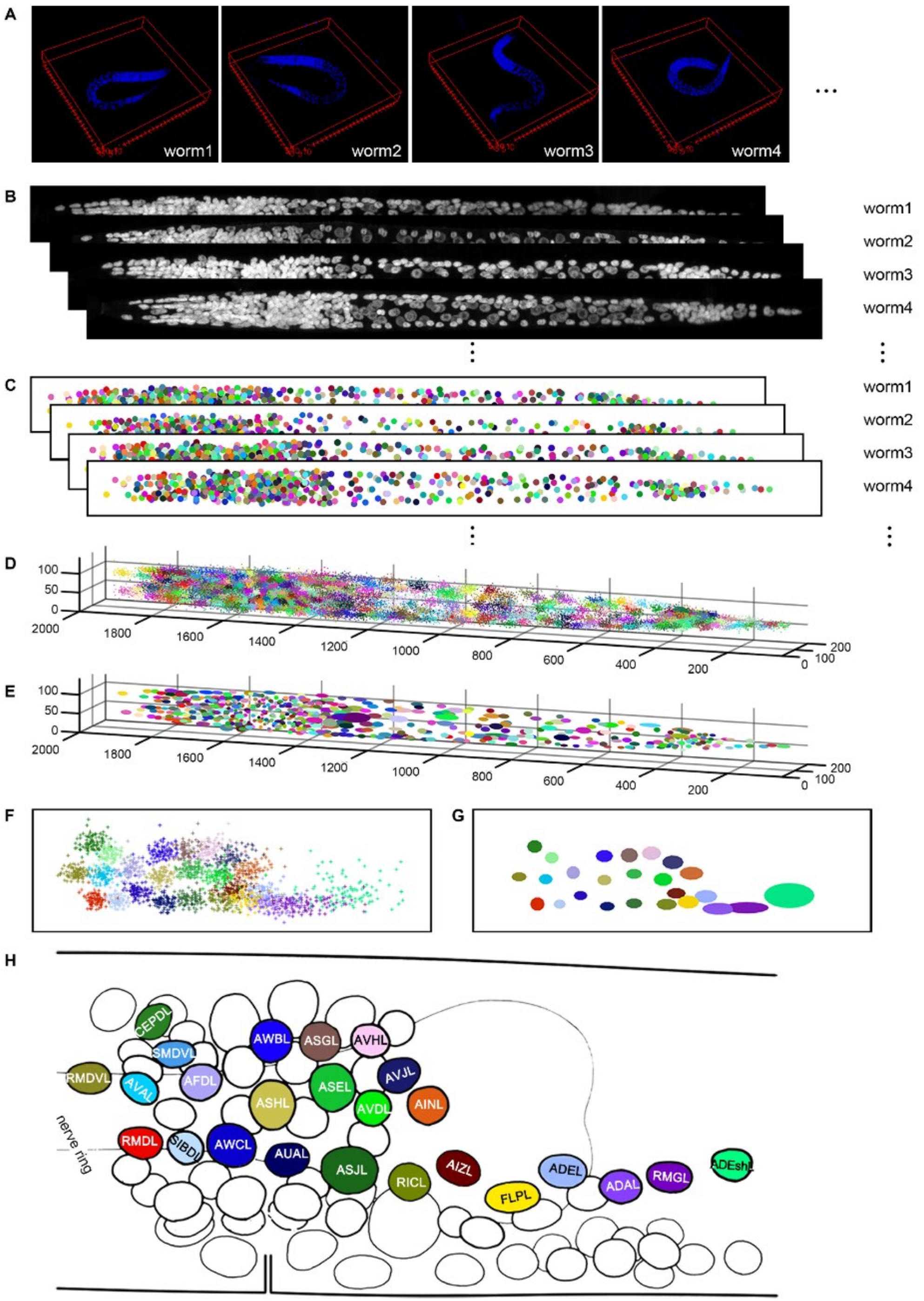

A
worm1
worm2
worm3
worm4
B
worm1
worm2
worm3
worm4
C
worm1
worm2
worm3
worm4
D
100
50
0
2000
1800
1600
1400
1200
1000
800
600
400
200
0
100
200
E
F
G
H
CEPDL
SMDVL
RMDVL
AVAL
AFDL
AWBL
ASGL
AVHL
ASEL
AVJL
ASHL
AVDL
AINL
nerve ring
RMDL
SIBDL
AWCL
AUAL
ASJL
RICL
AIZL
FLPL
ADEL
ADAL
RMGL
ADEshL

Supplementary Figure 1. Construction of a digital worm template of early stage L1 larvae. (**A**) Raw worm image stacks collected by confocal 3D scanning as training data. DAPI (blue) was used to stain all 558 nuclei. Shown are 2D (**B**) Annotated 3D worm image stacks. Shown are 2D slices of 3D worm image stacks straightened by the computation pipeline CellExplorer. The DAPI channel (grey) of these image stacks were segmented by CellExplorer to define nuclei and then their cell identities were manually annotated. (**C**) Mass centers of nuclei were extracted to represent the positions of all 558 nuclei in every training worm stack. Nuclei were labeled by pseudo-colors for visualization. Noticeably, information on nuclear size and morphology were not included in our worm template. (**D, F**) Registration of the 558 nuclei in 3D (**D**) or a set of neurons' nuclei in 2D (**F**) by aligning the training worm stacks to a worm stack chosen as initial target. (**F**) is color-coded for cell identity as per neuronal nucleus in (**H**). (**E, G**) The local point distribution model of our statistical atlases. Positional variability of 558 nuclei are represented as ellipsoids. (**H**) The well-established worm atlas used for manual cell identity assignment. Colored are neuronal nuclei shown in (**F, G**). The picture is adapted from previous published report [2].

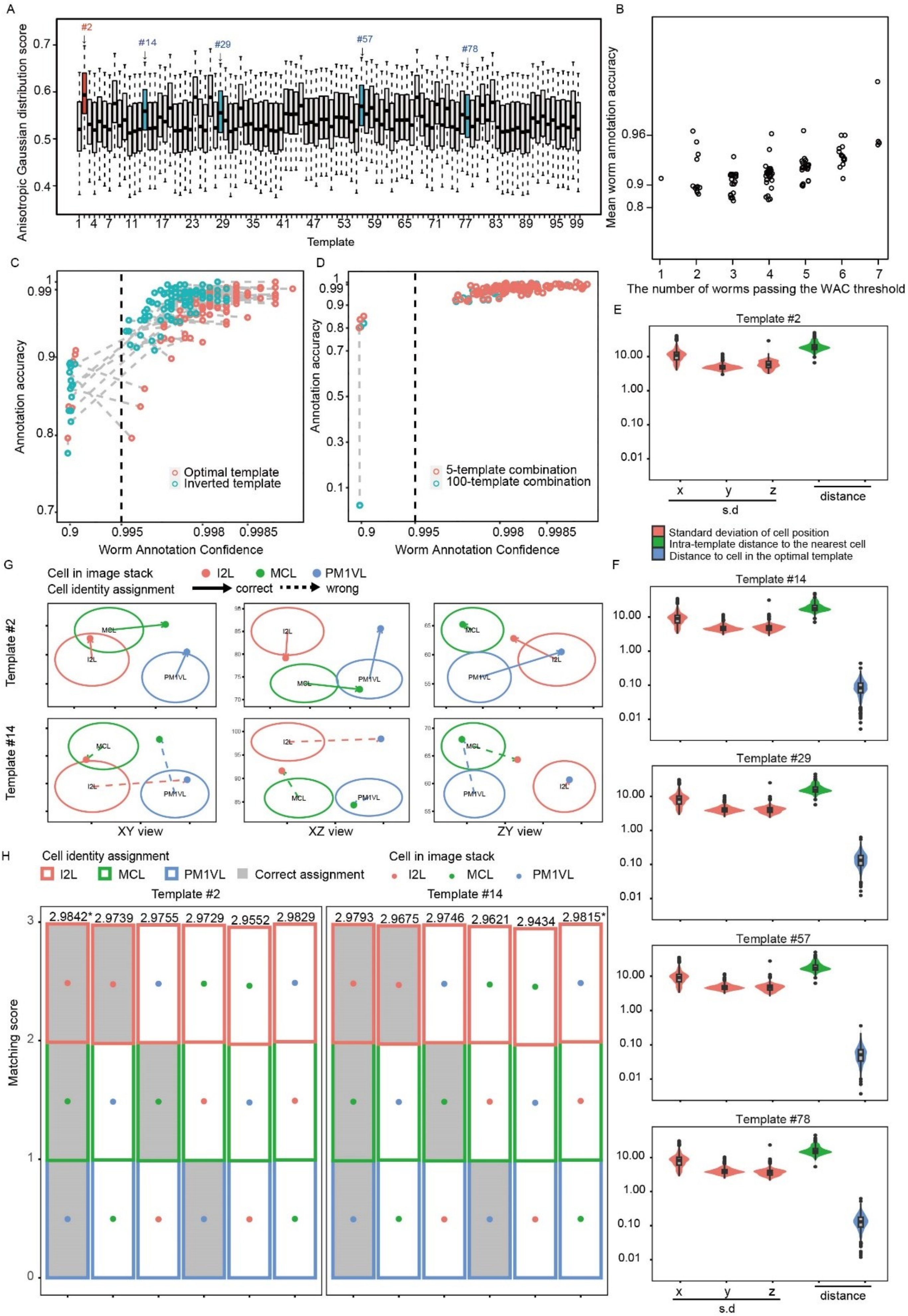
A
Anisotropic Gaussian distribution score
Template
#2
#14
#29
#57
#78
B
Mean worm annotation accuracy
The number of worms passing the WAC threshold
C
Annotation accuracy
Worm Annotation Confidence
Optimal template
Inverted template
D
5-template combination
100-template combination
E
Template #2
s.d
distance
Standard deviation of cell position
Intra-template distance to the nearest cell
Distance to cell in the optimal template
F
Template #14
Template #29
Template #57
Template #78
G
Cell in image stack
Cell identity assignment
I2L
MCL
PM1VL
correct
wrong
XY view
XZ view
ZY view
H
Cell identity assignment
Correct assignment
Matching score
2.9842*
2.9739
2.9755
2.9729
2.9552
2.9829
2.9793
2.9675
2.9746
2.9621
2.9434
2.9815*

Supplementary Figure 2. Selection of optimal and sub-optimal worm templates. **(A)** Fitness of each template to the 100 training worm stacks. Template #2 (red) fit the training worms best and was considered as the optimal template. Four sub-optimal templates (blue) were selected according to results in (**B**). Matching score is the Anisotropic Gaussian distribution score of fitting a template to a worm image stack. **(B)** Annotation accuracy of the 10 testing stacks by the 99 non-optimal templates. A point in the scatter plot represents a template. Every template was used to annotate the 10 testing stacks whose annotation based on the optimal template did not pass the 0.995 WAC cutoff. The most accurate annotation resulted from four templates (#14, #29, #57 and #78), in which seven testing stacks passed the 0.995 WAC cutoff. These templates that provided the best performance in annotating worm stacks that could not be readily annotated by the optimal template (blue) were then adopted as the alternative templates. (**C-D**) Accuracy of cell annotation and prediction power of WAC score at different modes of RAPCAT. Cell identity assignment by RAPCAT was compared with that of manual annotation to calculate annotation accuracy. In each plot, two annotation modes of same image stack were linked by a grey line. The vertical dashed lines represent the WAC cutoff to identify image stacks whose RAPCAT annotation result is highly reliable. Show are the second set of 100 testing worm stacks automatically annotated by RAPCAT, *i.e.*, before EPC. Orphan points were worm stacks with identical annotation resulted from two different modes. **(E-F)** Distribution of 558 cells in the optimal template (**E**) and each alternative template (**F**) with respect to their relative positions, including standard deviation in three coordinates, distance to the nearest cells, and distance to corresponding cells in the optimal template (#2) after affine transformation (only in **F**). Y-axes are in the unit of pixel. **(G-H)** One triple-cell case that an alternative template (#14) assigned correct cell identity while the optimal template (#2) did wrong. **(G)** Three cells are shown in three coordinates. Dots represent nuclear centers in an image stack after affine transformation**.** Ellipsoids represent positional variability of nuclear positions in templates. Cell names of ellipsoids are

shown. Arrows represent cell identity assignment by automatic RAPCAT based on corresponding templates. All axes are in the unit of pixel. **(H)** Bipartite cell identity assignment based on maximal matching score. Each stacked bar represents a triple-cell assignment, whose matching score is on top. asterisk, the chosen cell identity assignment based on given reference template.

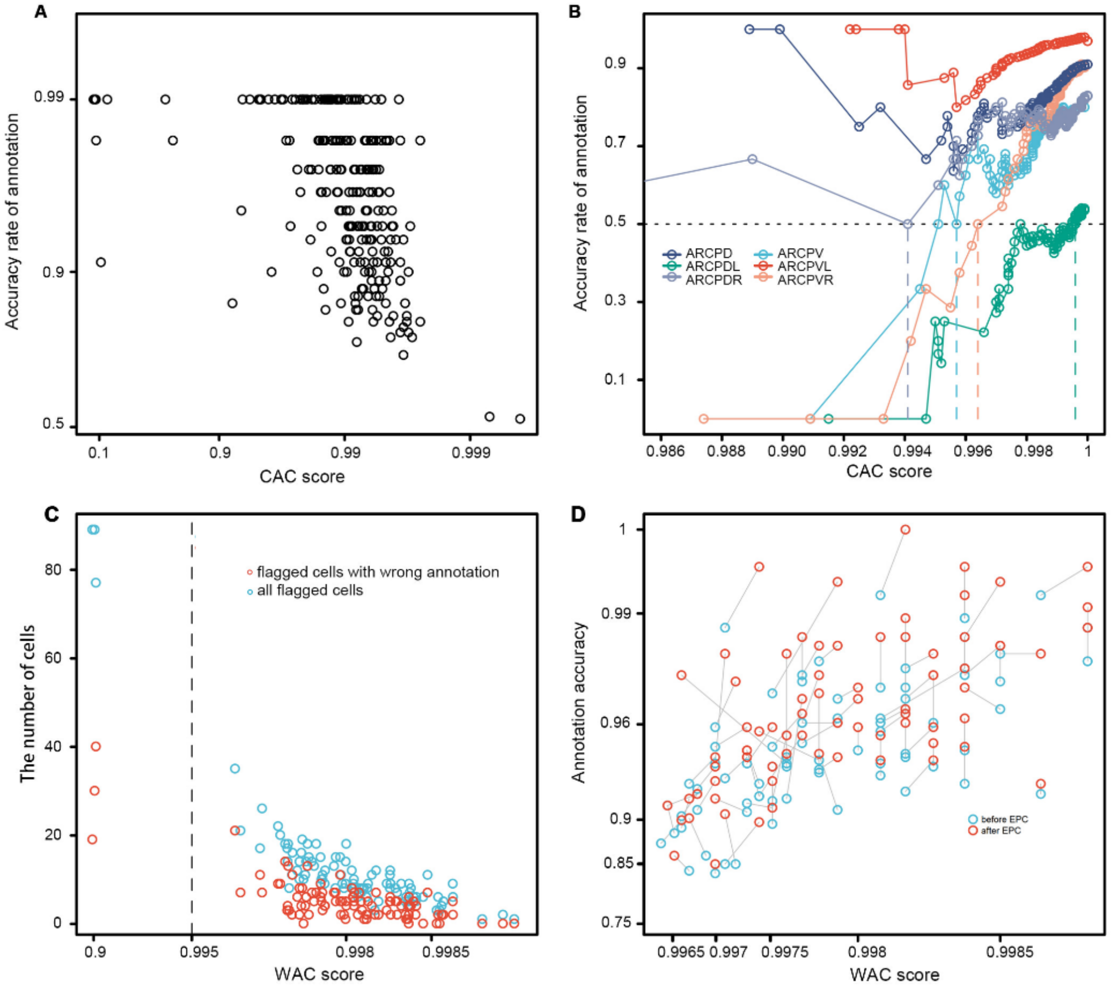
A
Accuracy rate of annotation
0.99
0.9
0.5
0.1
0.9
0.99
0.999
CAC score
B
Accuracy rate of annotation
0.9
0.7
0.5
0.3
0.1
ARCPD
ARCPDL
ARCPDR
ARCPV
ARCPVL
ARCPVR
0.986
0.988
0.990
0.992
0.994
0.996
0.998
1
CAC score
C
The number of cells
80
60
40
20
0
flagged cells with wrong annotation
all flagged cells
0.9
0.995
0.998
0.9985
WAC score
D
Annotation accuracy
1
0.99
0.96
0.9
0.85
0.75
before EPC
after EPC
0.9965
0.997
0.9975
0.998
0.9985
WAC score

Supplementary Figure 3. Various accuracy of RAPCAT cell annotation and prediction power of the CAC score. (A) Scatter plot showing that different cells had various accuracy rates of automatic annotation by RAPCAT in the first set of 100 testing worm image stacks. Each point corresponds to a cell identity. **(B)** Six posterior arcade (arcP) cells as examples of various prediction power of the CAC score for RAPCAT cell annotation. Each point represents a posterior arcade cell in one of the first set of 100 testing worm stacks. Cells of same identity in the 100 testing stacks are arranged according to their CAC scores and linked by lines. The Y-axis reflects the accuracy of RAPCAT annotation among cells with CAC no more than the given X value. Vertical dashed lines represent cell-specific CAC thresholds based on the 100 testing worm stacks. By definition, cells with CAC score no more than its specific threshold have 50% accuracy rate of automatic annotation. arcPD and arcPVL cells had no CAC threshold because their accuracy rates were more than 50% no matter which cutoff was used. **(C)** The prediction power of using cell-specific CAC thresholds for errors of RAPCAT cell annotation. Shown are the second set of 100 testing worm image stacks. Each worm is represented by a blue point and a red point. Flagged cells were those whose CAC scores were less than their cell-specific thresholds. Errors of cell annotation by RAPCAT were identified by manual examination. (**D**) Accuracy of cell annotation and prediction power of WAC score. Show are the 76 RAPCAT-annotated worm stacks in TF profiles before and after the EPC process

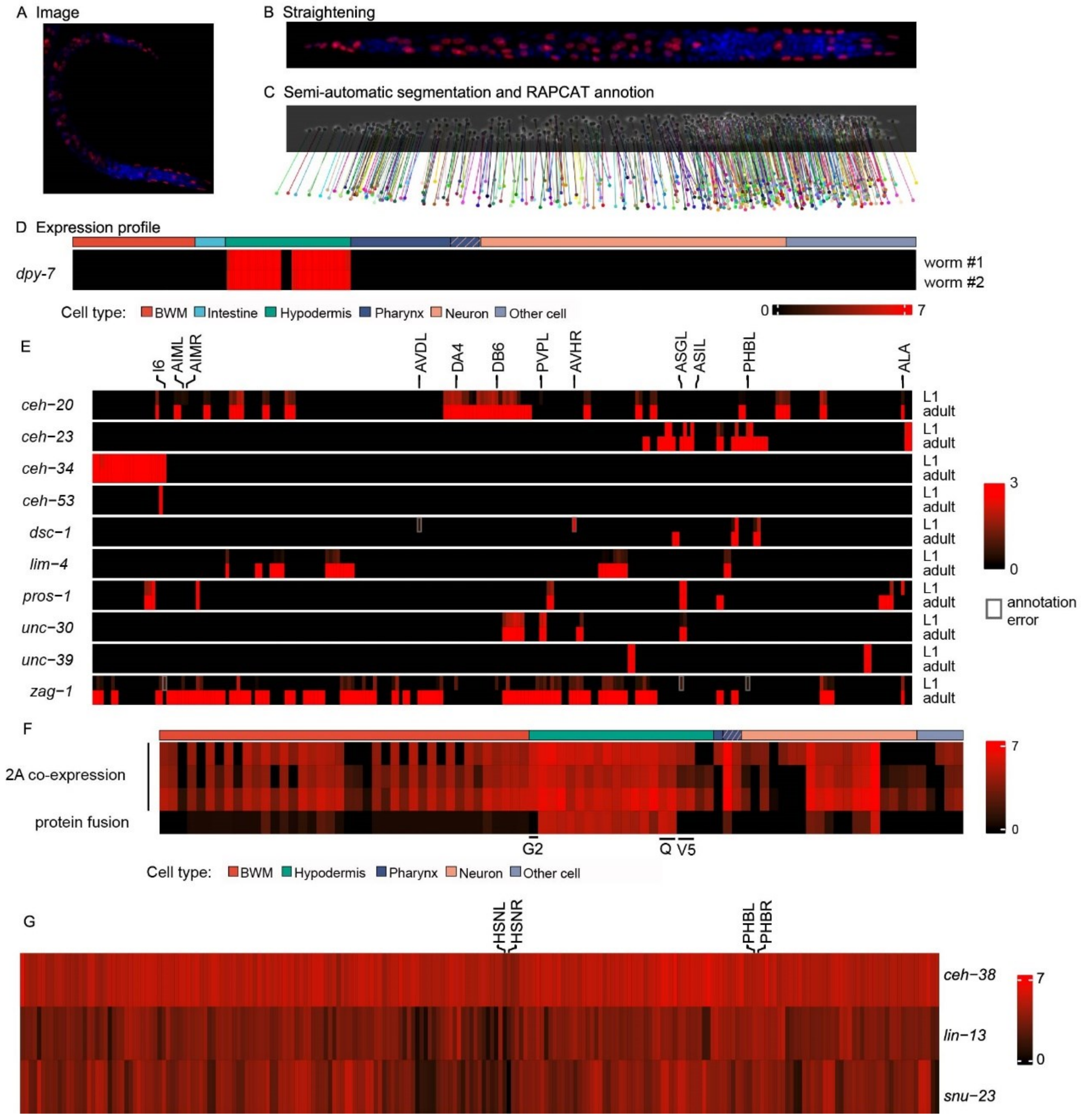
A Image
B Straightening
C Semi-automatic segmentation and RAPCAT annotion
D Expression profile
dpy-7
worm #1
worm #2
Cell type: BWM Intestine Hypodermis Pharynx Neuron Other cell
0
7
E
I6
AIML
AIMR
AVDL
DA4
DB6
PVPL
AVHR
ASGL
ASIL
PHBL
ALA
ceh−20
ceh−23
ceh−34
ceh−53
dsc−1
lim−4
pros−1
unc−30
unc−39
zag−1
L1
adult
3
0
annotation error
F
2A co-expression
protein fusion
G2
Q
V5
7
0
Cell type: BWM Hypodermis Pharynx Neuron Other cell
G
HSNL
HSNR
PHBL
PHBR
ceh−38
lin−13
snu−23
7
0

Supplementary Figure 4. Reporter expression profiling. (A-D) The pipeline of profiling reporter expression. (**A**) 3D image of a worm expressing a fluorescence reporter of interest and stained with DAPI. **(B)** the 3D confocal image is straightened by the computation pipeline CellExplorer. **(C)** Nuclei stained with DAPI are segmented by the CellExplorer and then automatically annotated by the RAPCAT. **(D)** Based on image output from the graphical user interface VANO, expression of the reporter in each of the 558 nuclei is calculated using custom MATLAB script and displayed as a heatmap. **(E)** Expression atlas of 10 homeodomain TF fosmid reporters in the 222 embryonically born neurons. The expression patterns in L4/young adults were previously published data [5] and qualitative (only on/off). Neurons are arranged in the same order as in Figure 2A. Names are shown only for the 12 neurons which expressed at least one of these 10 reporters at L1 stage, but not at young adulthood. All stacks were automatically annotated by RAPCAT, *i.e.*, before EPC step. **(F)** Expression profiles of two vab-15 knock-in reporters. Each row is a worm stack. Shown are cells that express at least one reporter. Cells are arranged in the same order as in Figure 2A. Cells addressed in the main text are labelled at the bottom of heatmap. **(G)** Expression atlas of broadly expressed protein fusion reporters. Neurons are arranged in the same order as in Figure 2A.

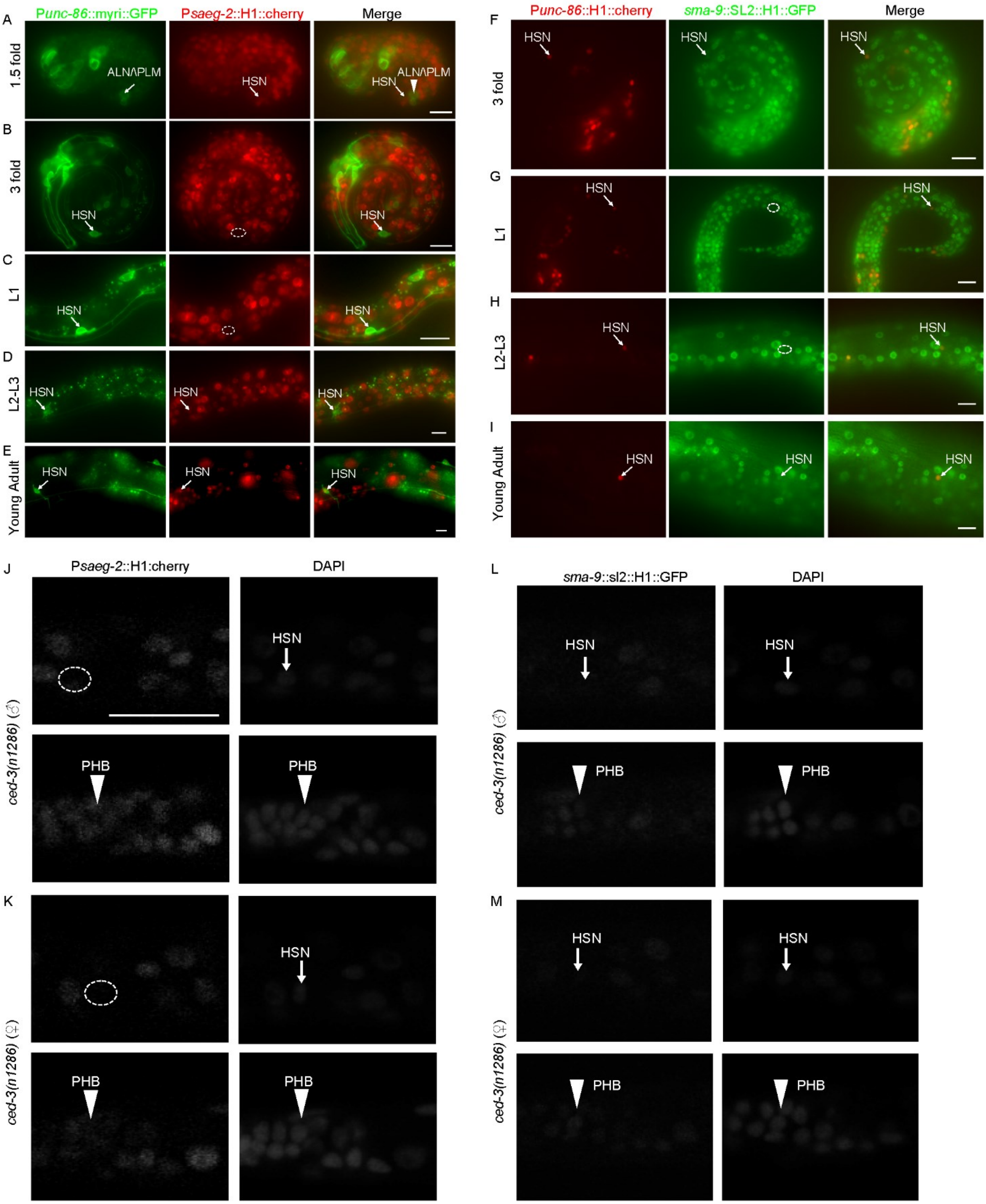
A
Punc-86::myri::GFP
Psaeg-2::H1::cherry
Merge
1.5 fold
ALN/\PLM
HSN
ALN/\PLM
B
3 fold
HSN
HSN
C
L1
HSN
HSN
D
L2-L3
HSN
HSN
HSN
E
Young Adult
HSN
HSN
HSN
F
Punc-86::H1::cherry
sma-9::SL2::H1::GFP
Merge
3 fold
HSN
HSN
HSN
G
L1
HSN
HSN
H
L2-L3
HSN
HSN
I
Young Adult
HSN
HSN
HSN
J
Psaeg-2::H1:cherry
DAPI
HSN
PHB
PHB
ced-3(n1286) (♂)
K
HSN
PHB
PHB
ced-3(n1286) (♀)
L
sma-9::sl2::H1::GFP
DAPI
HSN
HSN
PHB
PHB
ced-3(n1286) (♂)
M
HSN
HSN
PHB
PHB
ced-3(n1286) (♀)

Supplementary Figure 5 Temporal expression patterns of two TFs in HSN neurons during development. Shown are mosSCI cherry reporter transgene of *saeg-2* (**A-E, J**) and knock-in GFP reporter of *sma-9* (**F-I, K**). HSN neurons were annotated based on the expression of its marker gene *unc-86* in 3-fold embryos and during post-embryonic development. However, the *unc-86* GFP reporter had undetectable activity in any HSN neuron at 1.5 fold, right after its birth. So a newly born HSN was recognized based on its location relative the mother cell of ALN and PLM (ALN/PLM), which actively expresses *unc-86*. Statistics is in Figure 4I. (**J-M**) Shown are left-side of each L1 larva whose PHB and HSN neurons were photographed with same exposure time. Photos of each panel were taken from the same worm. The channel for *unc-86* marker is not shown. The sex of worm is determined by the position of coelomocyte cells [1]. 17 worms scored for (**J**, **K**), including 3 males, and 12 worms scored for (**L**, **M**), including 5 males. Arrows point to HSN neurons expressing reporter of interest while dot circles represent HSN neurons with little reporter activity. (Scale bar, 10 μm)

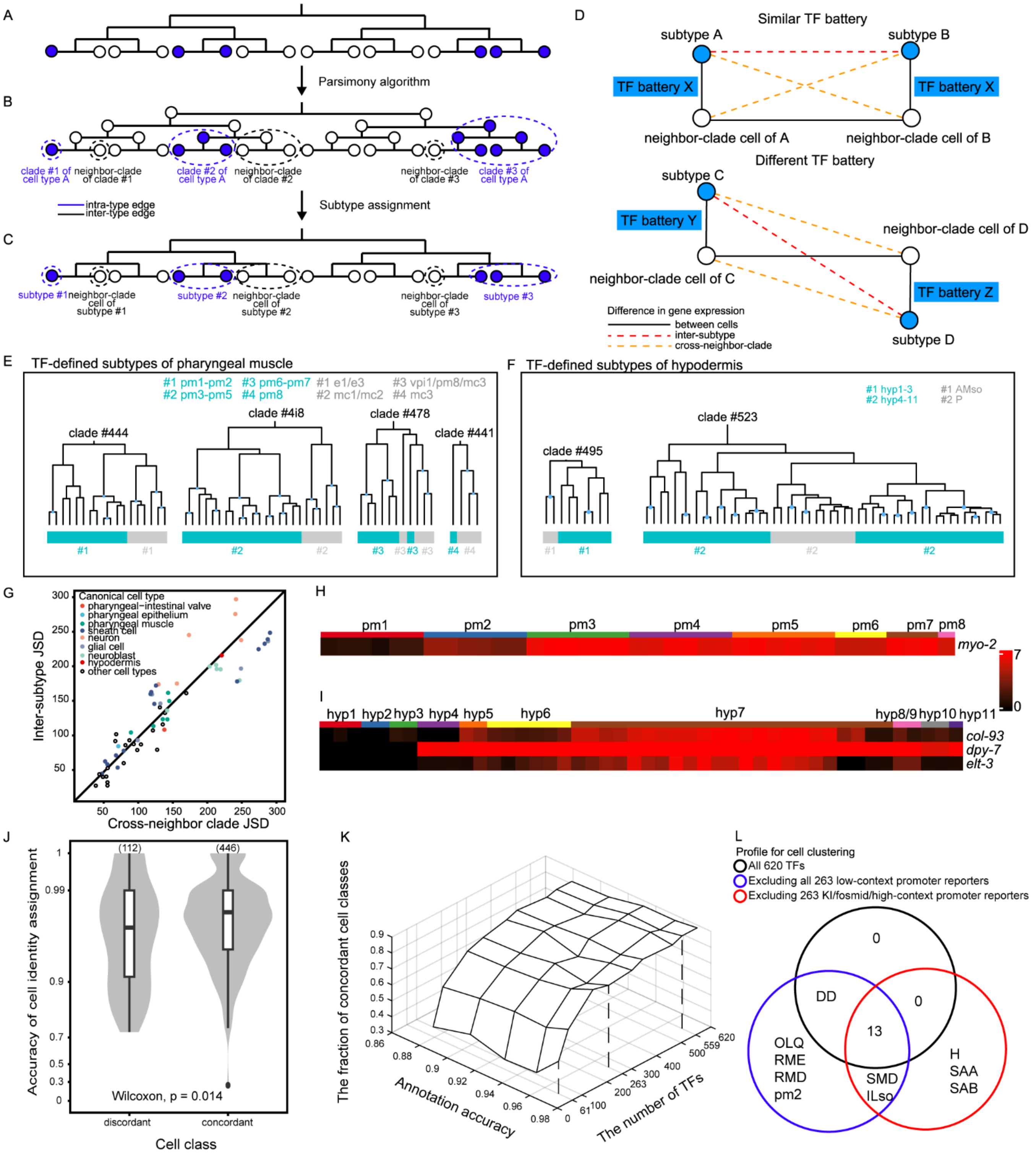
A
Parsimony algorithm
B
clade #1 of cell type A
neighbor-clade of clade #1
clade #2 of cell type A
neighbor-clade of clade #2
neighbor-clade of clade #3
clade #3 of cell type A
intra-type edge
inter-type edge
Subtype assignment
C
subtype #1
neighbor-clade cell of subtype #1
subtype #2
neighbor-clade cell of subtype #2
neighbor-clade cell of subtype #3
subtype #3
D
Similar TF battery
subtype A
subtype B
TF battery X
TF battery X
neighbor-clade cell of A
neighbor-clade cell of B
Different TF battery
subtype C
TF battery Y
neighbor-clade cell of D
neighbor-clade cell of C
TF battery Z
subtype D
Difference in gene expression
between cells
inter-subtype
cross-neighbor-clade
E
TF-defined subtypes of pharyngeal muscle
#1 pm1-pm2
#2 pm3-pm5
#3 pm6-pm7
#4 pm8
#1 e1/e3
#2 mc1/mc2
#3 vpi1/pm8/mc3
#4 mc3
clade #444
clade #4i8
clade #478
clade #441
F
TF-defined subtypes of hypodermis
#1 hyp1-3
#2 hyp4-11
#1 AMso
#2 P
clade #495
clade #523
G
Canonical cell type
pharyngeal−intestinal valve
pharyngeal epithelium
pharyngeal muscle
sheath cell
neuron
glial cell
neuroblast
hypodermis
other cell types
Inter-subtype JSD
Cross-neighbor clade JSD
H
pm1
pm2
pm3
pm4
pm5
pm6
pm7
pm8
myo-2
I
hyp1
hyp2
hyp3
hyp4
hyp5
hyp6
hyp7
hyp8/9
hyp10
hyp11
col-93
dpy-7
elt-3
J
Accuracy of cell identity assignment
(112)
(446)
Wilcoxon, p = 0.014
discordant
concordant
Cell class
K
The fraction of concordant cell classes
Annotation accuracy
The number of TFs
L
Profile for cell clustering
All 620 TFs
Excluding all 263 low-context promoter reporters
Excluding 263 KI/fosmid/high-context promoter reporters
DD
13
OLQ
RME
RMD
pm2
SMD
ILso
H
SAA
SAB

Supplementary Figure 6. Subtype classification by clustering gene expression profile. (A-C) Subtyping of a cell type by the TF profiles based on the distribution its cell members in a dendrogram. Blue leaf nodes represent cells of given type while white ones represent cells of not this type. The Parsimony algorithm assigns cell types (either 'given type' or 'not given type') to internal nodes to identify all independent clades of given cell type in the dendrogram (**B**). Leaf nodes of each clade represent an expression profile-defined subtype (**C**). (**D**) Types of gene expression differences between cell groups. Even if two subtypes have very similar molecular mechanisms underlying their differentiation from their respective neighbor-clade cells, they can be distantly related in terms of gene expression if their neighbor-clade cells have very different gene expression. The impact of gene expression difference between their neighbor-clade cells on inter-subtype gene expression difference can be computationally excluded by normalizing inter-subtype gene expression difference over cross-neighbor-clade gene expression difference. **(E,F)** Clades from the dendrogram showing TF-defined subtypes of pharyngeal muscle **(E)** and hypodermis **(F)**. Number sign in blue, TF-defined subtype; Number sign in grey, neighbor-clade cells. **(G)** Distance in TF expression between TF-defined subtypes. Each spot represents the inter-subtype (Y) and cross-neighbor clade value (X) between two subtypes. Color of a spot represents the phenotypic cell type which its two subtypes belong to. (**H,I**) Single-cell resolution reporter expression profile of pharyngeal muscle (**H**) and hypodermis(**I**). **(J-L)** The impact of technical limitations on the agreement between phenotype-based cell classification and TF-driven cell clustering. **(J)** The distribution of the 556 somatic cells in the concordant (single-clade in the dendrogram based on the 620-TF profiles) and discordant (spanning multiple clades) cell classes. Numbers in parentheses indicate cells. **(K)** The proportion of cell classes concordant between phenotype-based cell classification and cell clustering using specific TF profiles. Each group of worm stacks was annotated using five RAPCAT modes and the accuracy of each RAPCAT mode was determined using the second set of 100 testing stacks. The 61-TF profile

encompassed all fosmid/knockin reporters while the 263-TF profile encompassed all low-context promoter reporters. **(L)** A Venn diagram illustrating discordant cell classes based on various TF profiles.

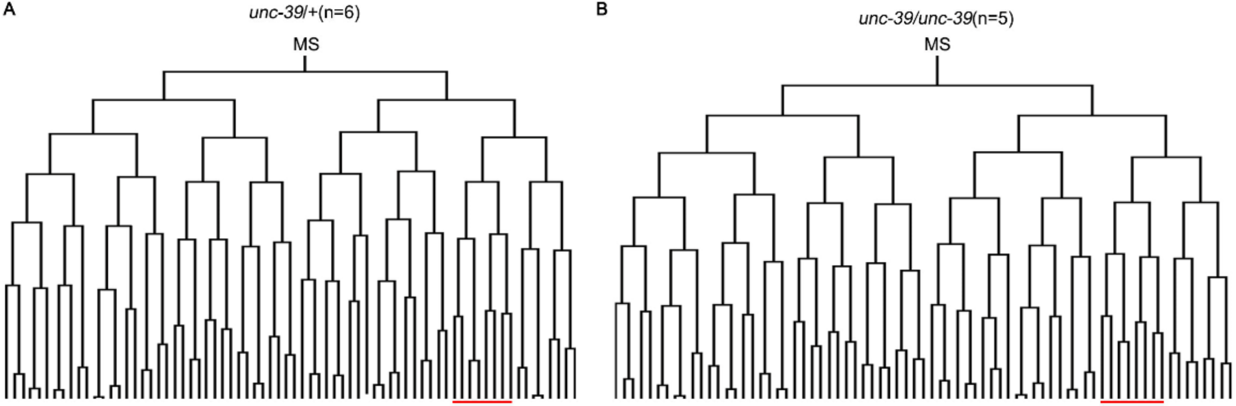
A
unc-39/+(n=6)
MS
B
unc-39/unc-39(n=5)
MS

Supplementary Figure 7 Cell division patterns of MS lineage. Scored were *unc-39* (*gk798*) heterozygous (**A**) and homozygous (**B**) embryos. The number of scored embryos were shown. Redbar, *unc-39*-expressing MS.(a/p)pa progeny.

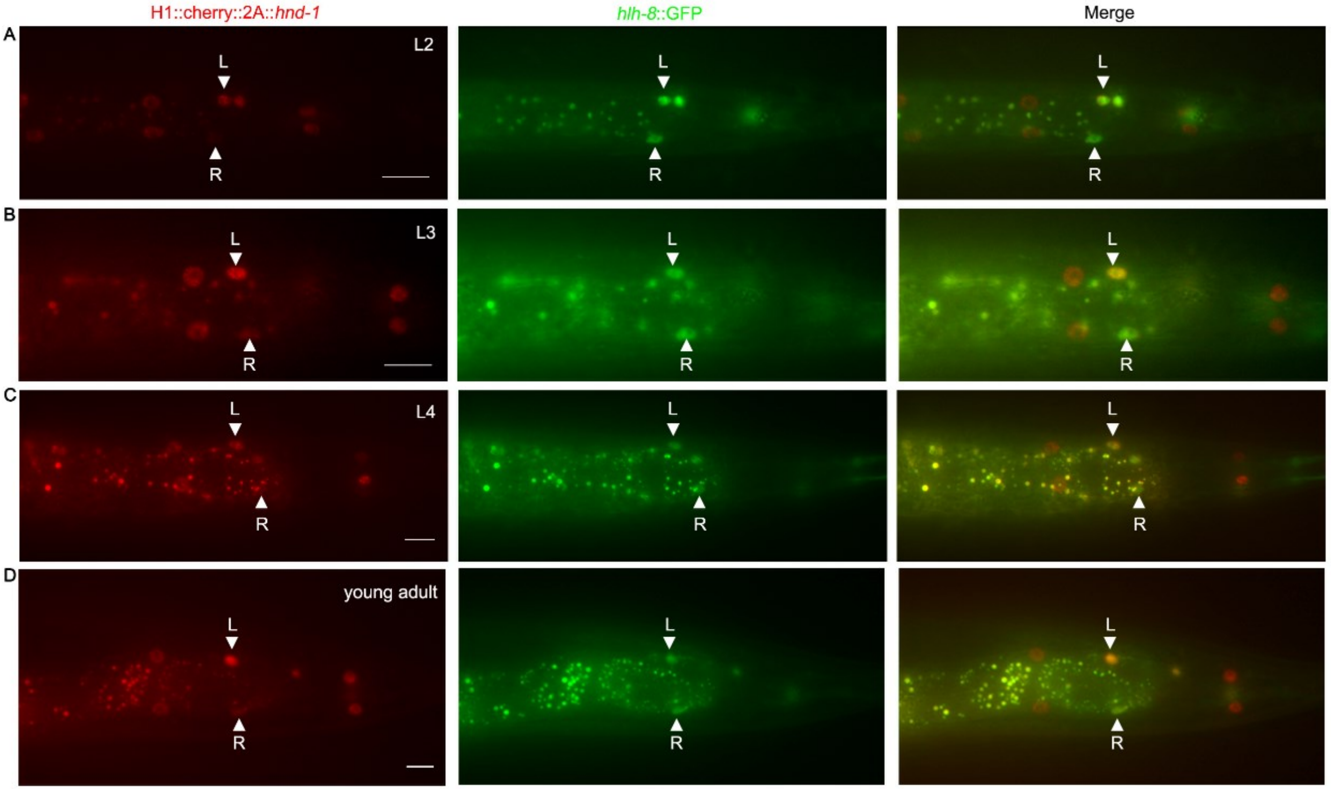
H1::cherry::2A::hnd-1
hlh-8::GFP
Merge
A
B
C
D
L2
L3
L4
young adult
L
R

Supplementary Figure 8 Temporal expression patterns of *hnd-1* KI reporter in intestinal muscles during post-embryonic development. Shown are ventral view of posterior regions of animals at L2 (**A**), L3 (**B**), L4 (**C**), and young adult (**D**) stages. The intestinal muscles are marked with arrowheads. Worms were oriented with left to the top. 20 animals were scored for each stage. (Scale bar, 10 μm) L, imL/AB.plpppppaa; R, imR/MS.ppaapp. The *hlh-8*::GFP reporter was used as a marker to label intestinal muscles.

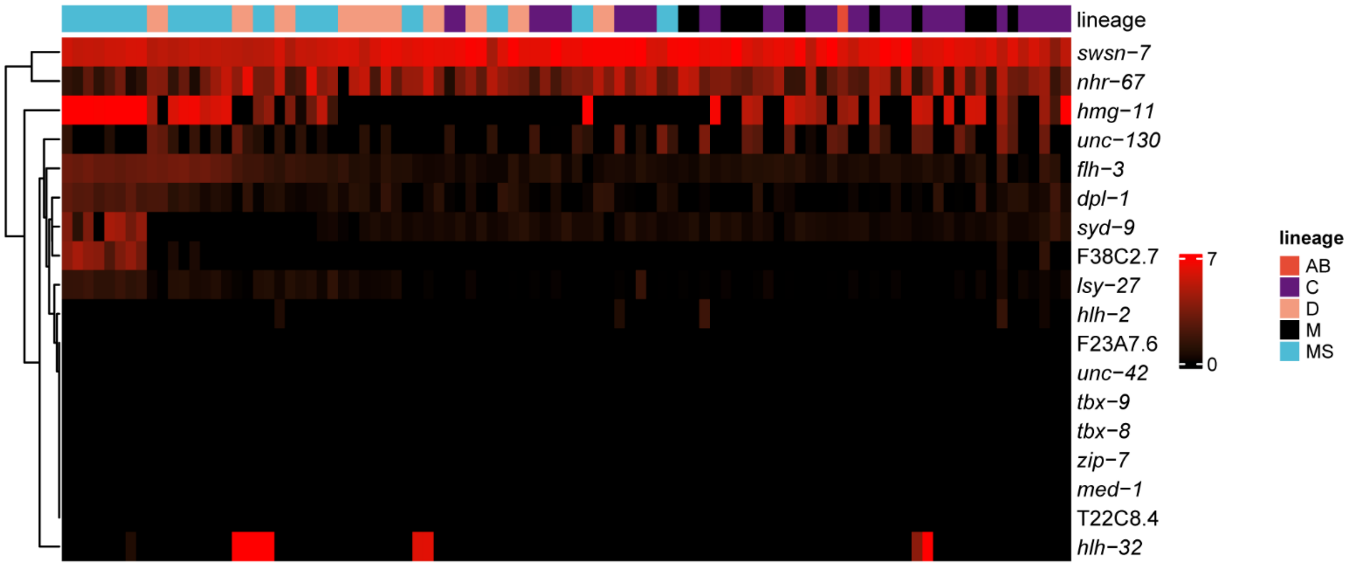
lineage
swsn−7
nhr−67
hmg−11
unc−130
flh−3
dpl−1
syd−9
F38C2.7
lsy−27
hlh−2
F23A7.6
unc−42
tbx−9
tbx−8
zip−7
med−1
T22C8.4
hlh−32
7
0
lineage
AB
C
D
M
MS

Supplementary Figure 9 Cell-lineage-related TFs in bodywall muscles at the stage of young adult stage. Bodywall muscles are arranged along anteroposterior axis.

Supplementary Table 1: Summary of HSN-specific low level reporter expression in HSN at L1 larval and young adult stages

Gene	Transgenesis	Reporter	Low expression in HSNs at L1	Low expression in HSNs at Young Adult
daf-3	Knock-in	sl2::cherry::H2B	10(14)	0(5)
dpff-1	Knock-in	sl2::H1::GFP	11(13)	0(5)
Y53G8AR.9	mosSCI	H1::cherry	11(15)	0(5)
Y56A3A.18	mosSCI	H1::cherry	12(15)	0(5)
F57A8.1	mosSCI	H1::GFP::2A	12(13)	0(5)
F57A8.1	Knock-in	H1::GFP	9(13)	0(5)
mxl-2	mosSCI	H1::cherry	9(12)	0(5)
nhr-49	mosSCI	H1::cherry	12(13)	0(5)
pqn-21	mosSCI	H1::cherry	10(14)	0(5)
saeg-2	mosSCI	H1::cherry	15(15)[a]	0(5)
sma-9	Knock-in	sl2::H1::GFP	11(15)	0(5)
zfp-1	mosSCI	H1::cherry	13(13)	0(5)

The number of scored animals are in parentheses.

Supplementary Reference

1. Sulston JE, Horvitz HR. Post-embryonic cell lineages of the nematode, Caenorhabditis elegans. *Dev Biol* **56**, 110-156 (1977).

2. Sulston JE, Schierenberg E, White JG, Thomson JN. The embryonic cell lineage of the nematode Caenorhabditis elegans. *Dev Biol* **100**, 64-119 (1983).

3. Yemini E*, et al.* NeuroPAL: A Multicolor Atlas for Whole-Brain Neuronal Identification in C. elegans. *Cell* **184**, 272-288 e211 (2021).

4. Peng H, Long F, Liu X, Kim SK, Myers EW. Straightening Caenorhabditis elegans images. *Bioinformatics* **24**, 234-242 (2008).

5. Reilly MB, Cros C, Varol E, Yemini E, Hobert O. Unique homeobox codes delineate all the neuron classes of C. elegans. *Nature* **584**, 595-601 (2020).

6. Sun H, Hobert O. Temporal transitions in the post-mitotic nervous system of Caenorhabditis elegans. *Nature* **600**, 93-99 (2021).

7. Albertson DG, Thomson JN. The pharynx of Caenorhabditis elegans. *Philosophical transactions of the Royal Society of London Series B, Biological sciences* **275**, 299-325 (1976).

8. Okkema PG, Harrison SW, Plunger V, Aryana A, Fire A. Sequence requirements for myosin gene expression and regulation in Caenorhabditis elegans. *Genetics* **135**, 385-404 (1993).

9. Altun ZF, Hall D, H. Epithelial system, hypodermis. In: *WormAtlas* (2009).

10. Gilleard JS, McGhee JD. Activation of hypodermal differentiation in the Caenorhabditis elegans embryo by GATA transcription factors ELT-1 and ELT-3. *Mol Cell Biol* **21**, 2533-2544 (2001).

11. Sandhu A, Badal D, Sheokand R, Tyagi S, Singh V. Specific collagens maintain the cuticle permeability barrier in Caenorhabditis elegans. *Genetics* **217**, (2021).

12. Whetstine JR, Ceron J, Ladd B, Dufourcq P, Reinke V, Shi Y. Regulation of tissue-specific and extracellular matrix-related genes by a class I histone deacetylase. *Mol Cell* **18**, 483-490 (2005).

13. Altun ZF, Hall DH. Excretory System. In: *WormAtlas* (2009).
