# Supplementary material for: A full-body transcription factor expression atlas with completely resolved cell identities in C. elegans
Source: Nat Commun. 2024 Jan 9;15:358. doi: 10.1038/s41467-023-42677-6 (PMC10776613; doi:10.1038/s41467-023-42677-6)
Supplement: Supplementary file 3 — Description of Additional Supplementary Files [file 41467_2023_42677_MOESM3_ESM.pdf]

Description of Additional Supplementary Files

Supplementary Data 1. Reporter strains for in situ TF expression profile

Supplementary Data 2. Expression profile of TF reporters in all 558 cells of L1 larvae

Supplementary Data 3. Cell types shared by three single-cell resolution datasets

Supplementary Data 4. Tissue-specific TFs

Supplementary Data 5. Reporter turn-off after 350-cell stage

Supplementary Data 6. Cell members of phenotypic cell types and TF-defined novel subtypes defined by TF profiling

Supplementary Data 7. Strain list for genetic analysis
