# Supplementary material for: A full-body transcription factor expression atlas with completely resolved cell identities in C. elegans
Source: Nat Commun. 2024 Jan 9;15:358. doi: 10.1038/s41467-023-42677-6 (PMC10776613; doi:10.1038/s41467-023-42677-6)
Supplement: Supplementary file 11 — Reporting Summary [file 41467_2023_42677_MOESM11_ESM.pdf]

nature portfolio

Corresponding author(s): Xiao Liu

Last updated by author(s): Oct 1, 2023

Reporting Summary

Nature Portfolio wishes to improve the reproducibility of the work that we publish. This form provides structure for consistency and transparency in reporting. For further information on Nature Portfolio policies, see our Editorial Policies and the Editorial Policy Checklist.

Statistics

For all statistical analyses, confirm that the following items are present in the figure legend, table legend, main text, or Methods section.

n/a	Confirmed	
☐	☒	The exact sample size (*n*) for each experimental group/condition, given as a discrete number and unit of measurement
☐	☒	A statement on whether measurements were taken from distinct samples or whether the same sample was measured repeatedly
☐	☒	The statistical test(s) used AND whether they are one- or two-sided<br>*Only common tests should be described solely by name; describe more complex techniques in the Methods section.*
☐	☒	A description of all covariates tested
☐	☒	A description of any assumptions or corrections, such as tests of normality and adjustment for multiple comparisons
☐	☒	A full description of the statistical parameters including central tendency (e.g. means) or other basic estimates (e.g. regression coefficient) AND variation (e.g. standard deviation) or associated estimates of uncertainty (e.g. confidence intervals)
☐	☒	For null hypothesis testing, the test statistic (e.g. *F*, *t*, *r*) with confidence intervals, effect sizes, degrees of freedom and *P* value noted<br>*Give P values as exact values whenever suitable.*
☒	☐	For Bayesian analysis, information on the choice of priors and Markov chain Monte Carlo settings
☐	☒	For hierarchical and complex designs, identification of the appropriate level for tests and full reporting of outcomes
☒	☐	Estimates of effect sizes (e.g. Cohen's *d*, Pearson's *r*), indicating how they were calculated

Our web collection on statistics for biologists contains articles on many of the points above.

Software and code

Policy information about availability of computer code

Data collection	Confocal image collection using Zen blue edition from Zeiss or Leica Application Suite.<br>Compound microscope image acquisition using Zen blue edition.
Data analysis	CellExplorer(https://github.com/lybCNU/CellExplorer)<br>VANO_v1.741<br>RAPCAT(https://github.com/lybCNU/RAPCAT)<br>ImageJ_1.50i<br>matlab_R2019a<br>philentropy_0.7.0<br>pvclust_2.2-0<br>ComplexHeatmap_2.12.1<br>ggplot2_3.4.1

For manuscripts utilizing custom algorithms or software that are central to the research but not yet described in published literature, software must be made available to editors and reviewers. We strongly encourage code deposition in a community repository (e.g. GitHub). See the Nature Portfolio guidelines for submitting code & software for further information.

Data

Policy information about availability of data

All manuscripts must include a data availability statement. This statement should provide the following information, where applicable:

- Accession codes, unique identifiers, or web links for publicly available datasets
- A description of any restrictions on data availability
- For clinical datasets or third party data, please ensure that the statement adheres to our policy

The worm image stacks generated in this study have been deposited in Zenodo (https://doi.org/10.5281/zenodo.7628038). The datasets utilized for training the digital worm templates, as well as the trained templates employed in this study, are available on GitHub and have been archived with Zenodo for long-term accessibility (https://doi.org/10.5281/zenodo.8399308). Source data are provided with this paper.

Research involving human participants, their data, or biological material

Policy information about studies with human participants or human data. See also policy information about sex, gender (identity/presentation), and sexual orientation and race, ethnicity and racism.

Reporting on sex and gender	N/A
Reporting on race, ethnicity, or other socially relevant groupings	N/A
Population characteristics	N/A
Recruitment	N/A
Ethics oversight	N/A

Note that full information on the approval of the study protocol must also be provided in the manuscript.

Field-specific reporting

Please select the one below that is the best fit for your research. If you are not sure, read the appropriate sections before making your selection.

☒ Life sciences ☐ Behavioural & social sciences ☐ Ecological, evolutionary & environmental sciences

For a reference copy of the document with all sections, see nature.com/documents/nr-reporting-summary-flat.pdf

Life sciences study design

All studies must disclose on these points even when the disclosure is negative.

Sample size	Sample size selection is based on past experience, preliminary data and power calculations. For example, the ability of molecular clustering to mirror established phenotype-based cell type classifications reaches a plateau when the profiles contain over 400 TFs. Our final profiles cover 620 worm TFs
Data exclusions	No data were excluded from analyses in the study.
Replication	Our biological findings and technological advancements are based on a minimum of two independent experimental replicates. Consistent protocols, reagents, and equipment settings were employed across these replicates. All attempts at replication were successful, confirming the reliability and reproducibility of our experimental findings.
Randomization	Given the nature of our study focusing on C. elegans inbreed lines with well documented developmental stages and homogeneous culture conditions, traditional allocation into experimental groups based on covariates was not deemed relevant.
Blinding	Investigators were blinded to group allocation during data collection. For example, worm annotators did not know gene names during cell name assignment before we compared our data with previously reported adult expression patterns of homeobox TF protein fusion reporters

Reporting for specific materials, systems and methods

We require information from authors about some types of materials, experimental systems and methods used in many studies. Here, indicate whether each material, system or method listed is relevant to your study. If you are not sure if a list item applies to your research, read the appropriate section before selecting a response.

Materials & experimental systems

n/a	Involved in the study
☒	☐ Antibodies
☒	☐ Eukaryotic cell lines
☒	☐ Palaeontology and archaeology
☐	☒ Animals and other organisms
☒	☐ Clinical data
☒	☐ Dual use research of concern
☒	☐ Plants

Methods

n/a	Involved in the study
☒	☐ ChIP-seq
☒	☐ Flow cytometry
☒	☐ MRI-based neuroimaging

Animals and other research organisms

Policy information about studies involving animals; ARRIVE guidelines recommended for reporting animal research, and Sex and Gender in Research

Laboratory animals	N2 and derivative strains of Caenorhabditis elegans at various developmental stages.
Wild animals	Not avaiable
Reporting on sex	hermaphrodite
Field-collected samples	The study did not involve samples collected from the field.
Ethics oversight	The study utilized the model organism Caenorhabditis elegans. Given the nature of this organism and the experiments conducted, no ethical approval or guidance was required.

Note that full information on the approval of the study protocol must also be provided in the manuscript.
