# Supplementary material for: A full-body transcription factor expression atlas with completely resolved cell identities in C. elegans
Source: Nat Commun. 2024 Jan 9;15:358. doi: 10.1038/s41467-023-42677-6 (PMC10776613; doi:10.1038/s41467-023-42677-6)
Supplement: Supplementary file 12 — Source Data [file Figure 3.pdf › Source Data/Figure 3/Figure 3.pdf]

Cluster dendrogram with p-values (%)

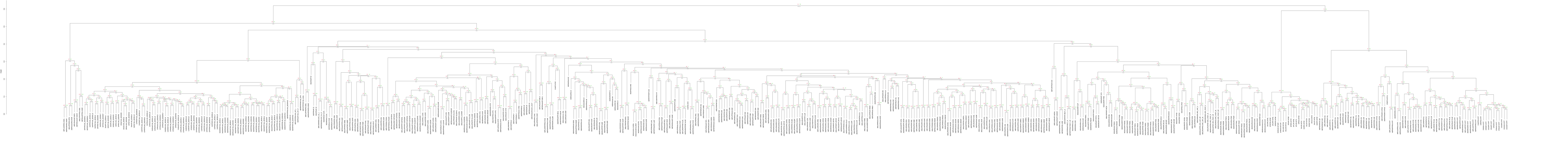

Distance: correlation
Cluster method: average
